# Platelet-Rich Plasma (PRP) Before Clinical Application: Qualitative Flow Cytometric Analysis and Enzyme-Linked Immunosorbent Assay (ELISA) Exploring Platelet Activation and TGFβ Release During Storage

**DOI:** 10.3390/biomedicines14020353

**Published:** 2026-02-03

**Authors:** Fulvia Costantinides, Violetta Borelli, Alvise Camurri Piloni, Lorenzo Bevilacqua, Michele Maglione

**Affiliations:** 1 Unit of Oral Surgery, School of Dental Sciences, Department of Medical, Surgical and Health Sciences, University of Trieste, Piazza dell’Ospitale 1, 34129 Trieste, Italy; 2 Department of Life Sciences, University of Trieste, 34129 Trieste, Italy; borelliv@units.it

**Keywords:** platelet-rich plasma, flow cytometry, ELISA, TGF-β

## Abstract

**Background/Objectives:** In clinical practice today, platelet concentrates are often used for topical surgical applications. They are biomaterials that can accelerate healing processes associated with oral and maxillofacial surgery as well as in several other clinical applications through the action of growth factors released by platelets at the surgical site. However, in most cases, the exact quantification of the released growth factors is challenging in both the short and long term. The aim of this study was to determine if early platelet activation and degranulation occur during the collection and utilization of platelet-rich plasma (PRP) in the surgical room, where, before its application, PRP undergoes a procedure of gelification via reactions with procoagulant agents. **Methods:** PRP was prepared from the blood samples of 39 patients following the modified Whitman protocol. The samples were then analyzed at four different time points (1, 6, and 24 h during preparation and clinical application in the surgery room) using flow cytometry and enzyme-linked immunosorbent assays (ELISAs) to investigate the platelet activation/degranulation and TGFβ release in the supernatant (SN) during storage and clinical application. The mean platelet count in the whole blood was 267.5 ± 48.58 × 10^3^/mL (range: 189–334 × 10^3^/mL), and the mean concentration was 2925.5 ± 833.37 × 10^3^/mL (range: 748–3453 × 10^3^/mL). **Results:** The activation and degranulation of platelet cells (measured via monoclonal antibodies: CD62p and CD63, respectively) demonstrated a progressive increase at 1 h, 6 h, 24 h, and after gelification. The TGFβ dosage in the supernatant (SN) at different times exhibited a similar trend, with a mean release of 18.36 ng/mL at 1 h, 21.96 ng/mL at 6 h, and 29.45 ng/mL at 24 h. After the gelification of the PRP, a significant reduction was observed, with a value of 15.52 ng/mL. **Conclusions:** The results reveal that the protocol used for the preparation, storage, and application of the PRP ensures a good-quality hemoderivative and that the platelet concentrate must be applied with the correct timing to support tissue healing processes.

## 1. Introduction

An important area in biomaterial research in recent years has been the local stimulation of healing after surgery. Platelets, leucocytes, fibrine matrices, and growth factors are involved in wound healing, working in synergy throughout the process. In order to improve surgical site healing, autologous and allogenous fibrin glues have been applied in clinical practice [1,2], followed by the use of platelet concentrates [3]. The concept behind platelet concentrates is based on blood centrifugation, which delivers a high number of platelets in a relatively small volume to a wounded site to ensure better and faster healing. Platelet concentrates are living biomaterials, and their clinical effects are dependent on the intrinsic versatile and adaptive characteristics of a patient’s blood [4]; their biology is as complex as that of blood.

Over the years, to create a biomaterial that is easy to manipulate during clinical applications, plasma activation (with autologous thrombin) has also been studied, achieving the fast polymerization of platelet-released fibrinogen in fibrin, leading to the formation of a platelet gel [5]. Despite various studies in different scientific fields and on different clinical applications, it has been difficult to ascertain the key role of each platelet concentrate component, such as fibrin and leukocytes [6,7,8], due to the complexity of the coagulation and healing process. However, among all components involved in modulating tissue healing, the growth factors released by platelets represent main actors [9].

The initial uncertainty regarding these materials caused a great deal of confusion surrounding correct terminologies in this field. The concept of platelet concentrate technologies initially emerged in oral and maxillofacial surgery following a publication by Marx et al. [10]. These authors wrote about “platelet-rich plasma (PRP)”, referring to platelet concentrates used in hematology for the treatment of sever thrombopenia. The concept of using platelet concentrates topically predates the article by Marx [11]. A few years earlier, Matras published research on fibrin glues [1], and autologous preparations named “platelet–fibrinogen–thrombin mixtures” were examined in ophthalmology [12,13], general surgery [14], and neurosurgery [15] contexts—these were also known as “gelatin platelets” [16]. Notably, these “platelet-rich products” were only tested as tissue adhesives, not as healing stimulators, considering the role of platelets as hemostatic/cicatrizing agents, and not as wound healing promoters via growth factor release.

In 1986, Knighton et al. [17] were the first to clinically demonstrate that platelet concentrates were able to promote healing locally. They wrote about “platelet-derived wound healing factors” (PDWHFs) obtained via a two-step blood centrifugation process, and they treated 49 patients with chronic non-healing cutaneous ulcers, achieving good outcomes. In other articles published in 1988 and 1990 [18,19], using a very different term, the same authors defined their product—the “platelet-derived wound healing formula (PDWHF)”—but they also used “platelet-rich plasma” when discussing transfusion medicine without referring to the final product. A few years later, the use of platelet concentrates in oral and maxillofacial surgery, obtained with a cell separator, was reported on by Whitman et al. [20]. During collection it was called PRP, but the final product was a fibrin gel and was named “platelet gel”. The exact content of all these products has never been completely investigated; only the platelets have been quantified.

The use of “PRP” as a term started with Marx et al. [10] in an initial study on the use of these platelet-rich products during bone graft reconstruction in maxillofacial surgery. The clinical results of the bone healing were very interesting and encouraged the use of these technologies in this field. Over the following years, many studies tested various in-house protocols and modified the basic two-step centrifugation process, testing different centrifugation times (from 3 to 20 min for the different centrifugation steps) and forces (from 160× *g* to 3000× *g*). All these parameters seem to have been empirically defined, and the products were poorly described and characterized, particularly in regard to their cellular content.

To date, many studies have quantified the growth factor content of these products/PRP; however, without considering the platelet activation steps, it is difficult to achieve accurate conclusions [21,22].

Studies on animal models, in vitro experiments, and clinical trials have evaluated the biological behavior of this grafting material using different PRP preparation and activation techniques [10,20,23,24,25,26,27]. However, the available data are controversial regarding the improvement of bone regeneration in oral and maxillofacial surgery owing to the biological action of PRP. Studies by Marx et al. [10] demonstrated that using PRP, in combination with autologous bone, induces faster bone maturation (radiographic evaluation) and the regeneration of bone tissue with an augmented density (histomorphometry evaluation). On the other hand, Freymiller & Anghaloo highlighted that there is a lot of conflicting data in the PRP literature regarding its use alone or in combination with inorganic bone minerals or organic bone matrices. The authors hypothesized that the technique used for PRP collection and storage is important and may contribute to early and premature platelet degranulation.

The aim of this study was to investigate the processes of platelet activation and degranulation during the collection of platelet-rich plasma in order to optimize its utilization in the surgical room. A flow cytometry analysis of platelet functionality and the dosage of growth factors in the supernatant (SN) was performed to quantify the platelet activation before clinical utilization and gelification via procoagulant agents.

## 2. Materials and Methods

### 2.1. Population

Thirty-nine patients (mean age 37.62 ± 16.84) requiring extractions of included teeth or cyst enucleation at the Unit of Oral Surgery of the School of Dental Sciences (University of Trieste) were selected; the study was conducted according to the Declaration of Helsinki and with the approval of the ASUGI ethics committee (number 0013567/P/GEN/ARCS, 6 April 2022). The exclusion criteria were coagulation alterations, the use of anti-thrombotic agents, and all hematic and metabolic disorders that could interfere with the healing of residual cavities. Patients with uncontrolled diabetes, chronic renal insufficiency, pregnancy, acute and chronic hepatic diseases, and immunodepression were not selected for this study. The coagulation tests assessed the platelet count, PT (pro-thrombin time), INR (Index Normalization Ratio), and APTT (activated partially thromboplastin time). Informed consent forms were distributed and completed by each patient before blood was drawn.

### 2.2. Blood Collection

The blood preparation procedure was carried out by the Immunotransfusional Service of the “Maggiore” Hospital (ASUGI, Trieste, Italy). From each patient, 400 mL of blood was drawn the day before surgery to obtain a 15 mL aliquot of PRP. Samples were collected using an automated plasmapheresis system along with a closed and sterile plastic tubing set containing a citrate anticoagulant, and the blood was centrifuged twice (Cryofuge 6000i, Controltecnica Instrumentatión Cientifica, S.L., Madrid, Spain) at a temperature of 20 °C. After the first cycle (100× *g* for 13 min), the red cells (that could be reinfused) were separated from the residual plasma. The residue, containing platelets, underwent a second centrifugation (1340× *g* for 18 min) that dissociated the platelet concentrate, or PRP, (about 15 mL) from the fresh plasma. Then, 10 mL of plasma was mixed with 3 mL of 6% calcium gluconate and incubated for 30 min at 37 °C. By squeezing the residual clot, autologous thrombin (AT) was obtained and collected in a transfer pack. All steps were carried out using Terumo^®^ sterile connectors (Terumo Corporation, Tokyo, Japan). Cryoprecipitate was obtained from the residual plasma through a rapid freezing and subsequent defrosting procedure at 4 °C. Cryoprecipitate is a concentrate of high-molecular-weight plasma proteins that precipitates in the cold. This concentrate contains factor VIII, von Willebrand factor (vWF), fibrinogen, factor XIII, and a few other cryoprecipitable proteins, including fibronectin [1]. The collected PRP was left to rest for 1 h on a laboratory agitator (t° 22 °C) in order to obtain a homogenous dispersion of the platelets in the bag.

### 2.3. Study Design/PRP Sample Preparation

A 30 cm long (diameter 5 mm) connector was attached to the bottom side of the PRP container, allowing a small amount of PRP to flow inside. The connector was then sealed and detached from the main container. The whole connector was subdivided into 3 units using a dedicated device. All units contained approximately 500–750 μL of sterile platelet-rich plasma. Samples were left to agitate near the main PRP container to guarantee the same treatment conditions

All storage units were opened after 1, 6, and 24 h of agitation, and 200 µL was transferred to a test tube, immediately fixed with the same (or 1:1) amount of ThromboFix platelet stabilizer (Immunotech Coulter, Westbrook, ME, USA), and stored at room temperature until the flowcytometric analysis. The residual PRP was centrifuged for 10 min at 2490× *g*, the platelets were separated, and the supernatants were frozen at −80 °C until the ELISA.

Our analysis at the 24 h mark was based on the fact that, typically, the day after PRP preparation, bags are transferred to the surgical room for gel preparation and application during maxillofacial surgery.

As noted by Barbero and colleagues [28], it is difficult to apply PRP at the surgical site as a pellet after centrifugation because of its lack of consistency. To obtain a manipulable blood component, the gelification procedure must be performed in the surgery room. The Whitman protocol calls for a mix of the cryoprecipitate (2 mL), calcium gluconate (1 mL), and autologous thrombin (2 mL) for every 7 mL aliquot of PRP. The gel results in a filamentous network in which platelets are bridled and start to release growth-factor-containing alpha granules due to the stimulus of calcium gluconate and autologous thrombin [28].

### 2.4. Flow Cytometry Analysis

Each sample, fixed and stabilized with the TromboFix Platelet Stabilizer^®^ (Beckman Colter, Brea, CA, USA), was labeled with the following monoclonal antibodies:

- CD42 Fitc (Beckman Colter, Brea, CA, USA): to identify and select the platelet population based on the presence of this specific membrane receptor.
- CD62p Pe (Beckman Colter, Brea, CA, USA): to highlight the percentage of activated cells.
- CD63 Pe (Beckman Colter, Brea, CA, USA): to highlight the percentage of degranulated cells.

For this purpose, two aliquots of PRP, 20 μL for each collection condition (collected after 1, 6, and 24 h of the preparation of the PRP and at the time of its gelification), were labeled with (a) 10 μL of CD62p Pe (CD42-Fitc/CD62 Pe) or (b) 10 μL of CD42 Fitc and 10 μL CD63 Pe (CD42-Fitc/CD63 Pe), respectively, (double labeling with two different marker pairs for each sample).

All labeled samples were incubated for 30 min in the dark at room temperature and were then washed with 1 mL of PBS (Phosphate-Buffered Solution) to eliminate any unbound antibodies and the fixative-stabilizing solution.

After centrifugation at 708× *g* for 10 min at room temperature, the supernatant was removed, and the pellet was resuspended in 1 mL of the PBS. For the sample reading and analysis, an Epics Elite ESP (Coulter, Miami, FL, USA) with an Interprise laser was used, with emissions at 488 nm.

The following parameters were selected:

- Frontal Scatter (FS): to highlight cell dimensions;
- Side Scatter (SS): to highlight the cellular grain;
- Photomultiplier 2 (PMT2) for the acquisition of the fluorescence at 525 nm corresponding to the emission band of the Fitc associated with the platelet marker CD42;
- Photomultiplier 3 (PMT3): for the acquisition of fluorescence at 575 nm corresponding to the emission band of the Pe associated with the CD62p platelet activation and CD63 degranulation marker.

**Figure 1:**
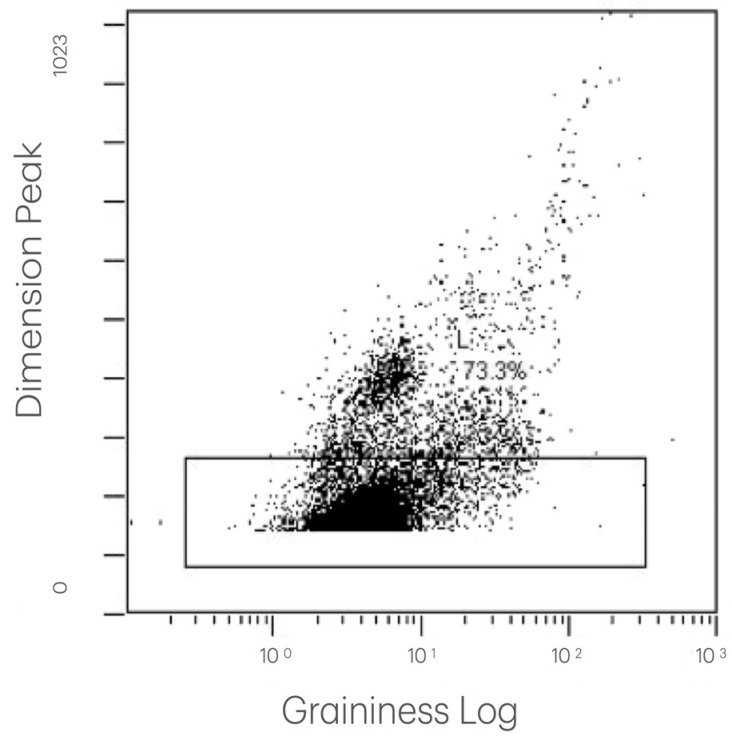
The distribution of particles in a representative PRP sample. This dot plot displays particles measured by flow cytometry, with the granularity on the X-axis (log scale) and the particle size (dimension peak) on the Y-axis. Each point represents a single particle. The plot enables the visualization of different populations based on their size and internal complexity. It illustrates the distribution of particles and supports the gating strategy used in this study.

**Figure 2:**
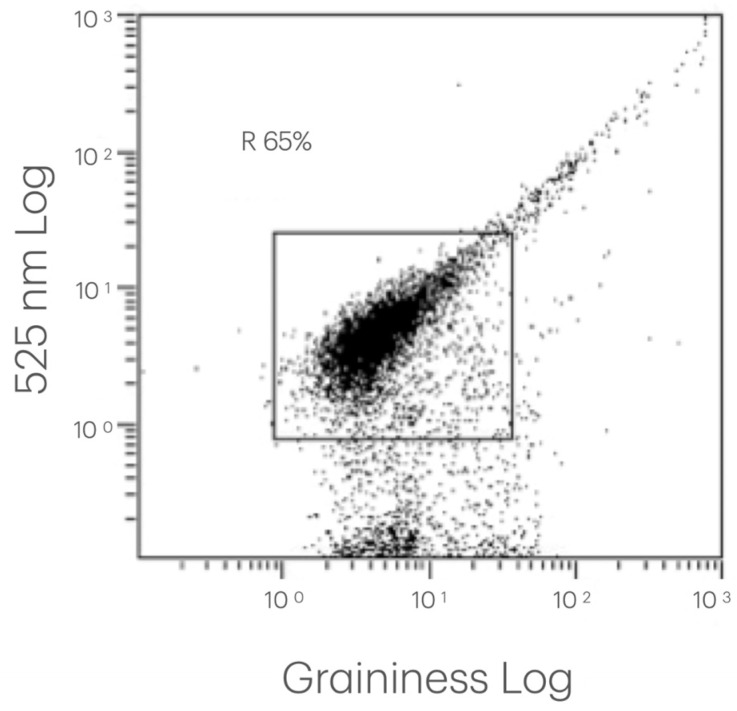
Identification of platelet population (CD42) in CD42-labeled PRP analyzed via fluorescence flow cytometry. Representative dot plot diagram of an individual PRP sample, displaying particles measured using flow cytometry, with granularity on the X-axis (log scale) and fluorescence at 525 nm (CD42-FITC) on the Y-axis (log scale). Each point represents a single particle, allowing us to distinguish FITC-positive (CD42 positive) and FITC-negative (CD42 negative) populations, independently of granularity.

**Figure 3:**
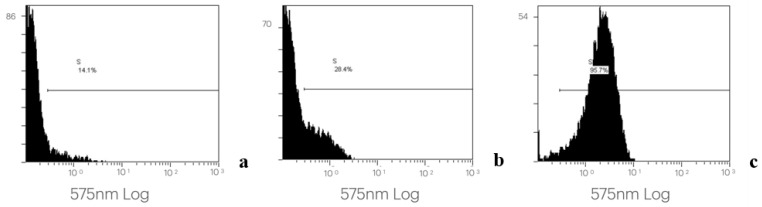
The expression of platelet membrane antigens CD42 and CD62 in PRP at different time points: 1 (**a**), 6 (**b**), and 24 h (**c**). Platelets are double-labeled (CD42 FITC-CD 62) for identification of active cells. Cell count/fluorescence intensity histograms of one representative PRP sample.

The collected and processed data allowed us to identify and quantify the platelet population and, within gate CD42/SS, to verify the changes in the expression of platelet activation and degranulation markers both over time and between patients. Examples of the flow cytometry data acquisition are presented in Figure 1, Figure 2 and Figure 3.

### 2.5. Measurement of TGF Levels via Enzyme-Linked Immunosorbent Assay

An enzyme-linked immunosorbent assay (ELISA) was performed to quantify the amount of TGFβ1 in the supernatants and pellets at each time point.

**Figure 4:**
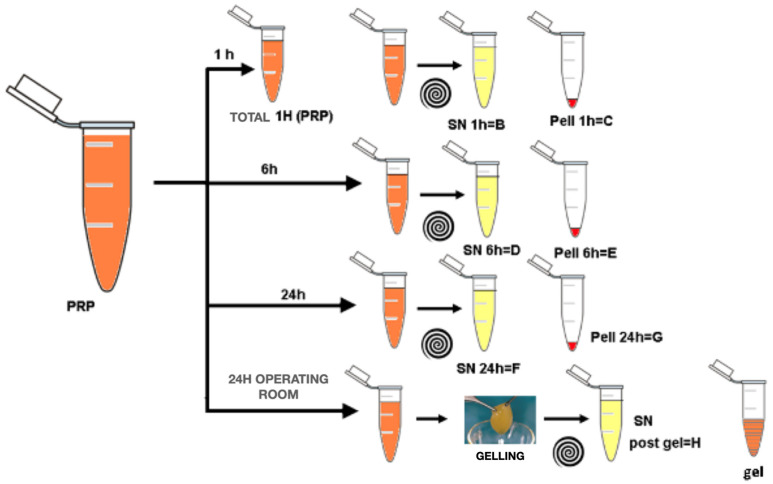
Description of PRP partition and analysis at different times.

After defreezing, the pellets were resuspended in a volume of the buffered physiological solution that was equal to that of the supernatant, and all the samples were subjected to a sonication process in order to release the platelet-enclosed TGFβ. Then, they were centrifuged (2780× *g* for 10 min at room temperature) to eliminate the particulate, and the supernatant was dosed with the ELISA TGFβ1 Emax (Promega, Madison, WI, USA). The workflow is presented in Figure 4. The TGFβ concentration was measured accordingly to the manufacturer’s instructions. Briefly, to ensure that TGFβ bound to the antibody (and thus to evaluate its bioactive form), we activated it via acidification with 1N HCl and subsequently neutralized the solution with 1N NaOH, as suggested by the manufacturer. Optimal dilutions for each PRP fraction (total, pellet, and supernatant) have been preliminarily established by dilution curves and were, respectively, 1:300, 1:150, 1:600, and 1:1200 for the SN, total, and pellet fractions. Triplicates were performed for all assays.

The TGFβ levels in the SN were expressed as mean values and as percentage release values % with respect to the total amount of TGFβ contained in the sample. The following calculation was performed:

% release = (TGFβ concentration in the SN)/(TGFβ concentration in the total)

### 2.6. Statistical Analysis

The statistical analysis was performed with the SPSS program v 18.0 for Mac Os (SpSS Inc., Chicago, IL, USA) using the Friedman test. Significance was denoted by a *p*-value of 0.05.

## 3. Results

The mean platelet count in the whole blood was 267.5 ± 48.58 × 10^3^/mL (range: 189–334 × 10^3^/mL), and in the PRP it exhibited a mean concentration of 2925.5 ± 833.37 × 10^3^/mL (range: 748–3453 × 10^3^/mL).

**Figure 5:**
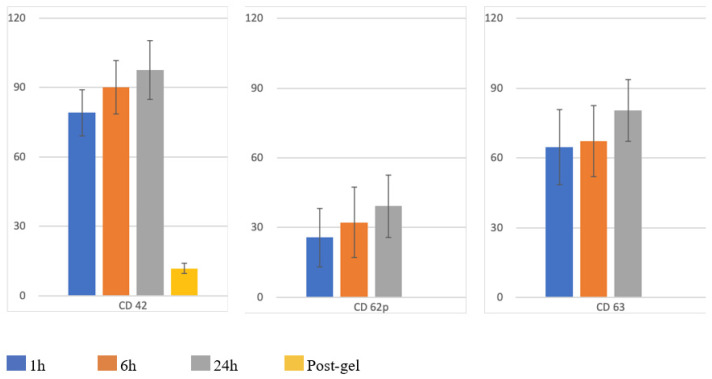
Expression levels of platelet markers CD42, CD62p, and CD63. The bar chart illustrates the percentage of positive cells for the indicated markers at different time points (1 h, 6 h, 24 h, and post-gel). Blue bars represent 1 h values, orange bars represent 6 h, gray bars represent 24 h, and the yellow bar indicates the post-gelation phase. Data are expressed as mean ± standard deviation (SD).

The results of the flow cytometry analysis are presented in Figure 5 and Table 1.

CD42 exhibited a mean percentage of 79.1% at 1 h, 90.2% at 6 h, 97.6% at 24 h, and 11.9% after gelification. The range of the CD62p positivity (platelets activation) was 0.2–70.4% (mean: 25.71% ± 25.60%) at baseline (1 h), 0.3–74.1% (mean: 32.25% ± 30.85%) at 6 h, and 0.4–71.6% (mean: 39.21% ± 27.48%) at 24 h. The range of degranulated platelets (CD63) was 16.1–94.2% (mean: 59.86% ± 32.75%) at the baseline, 15.4–96.6% (mean: 62.99% ± 31.02%) at 6 h, and 60.8–98.9% (mean: 80.43% ± 16.94%) at 24 h.

Independently from the baseline value for CD62p and CD63, a continuous increase in percentages was observed for all samples over time.

**Figure 6:**
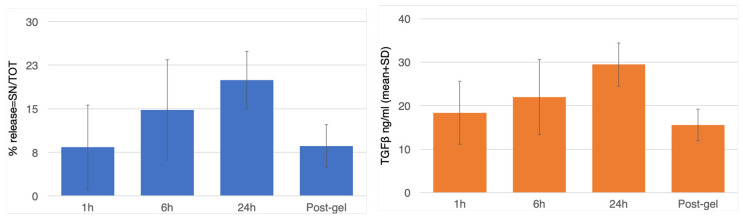
TGFβ release from platelets at different time points. Values are expressed as percentage (% release = TGFβ concentration in the SN/TGFβ concentration in the total) and as mean concentration in the SN of TGFβ ng/mL ± SD.

A similar trend was observed in the release of TGFβ in the supernatant at different time points, with a mean TGFβ release of 18.36 ± 12.41 ng/mL at 1 h, 21.96 ± 13.15 ng/mL at 6 h, and 29.45 ± 14.24 ng/mL at 24 h. After gelification, the values decreased to 15.52 ± 24.24 ng/mL (Figure 6 and Table 2).

## 4. Discussion

As described in the Introduction, platelet-rich plasma (PRP) was introduced in the literature by Whitman et al. in 1997 [20], and following the research by Marx et al. [10], PRP immediately attracted high levels of interest in the scientific community, becoming a subject of extreme relevance in dental and maxillofacial practice [29,30,31,32]. The biological potential of PRP depends not only on its hemostatic properties but also on the presence of numerous biomolecules that stimulate tissue metabolism. Indeed, platelet-rich plasma, obtained via the collection of autologous whole blood and the separation of its constituents [10,33], represents an autologous source of growth factors (TGF-β_1_, TGF-β_2_, VEGF, EGF, IGF, PDGF-αα, PDGF-ββ, and PDGF-αβ). These growth factors have been proven to be chemotactic for cells migrating into skin wounds undergoing healing, such as neutrophils monocytes and fibroblasts, and to be important for cell differentiations and matrix deposition [28,34,35]. Furthermore, PRP contains fibrin, fibronectin, and vitronectin—three blood proteins that increase cellular adhesion and create a matrix for the migration of epithelial cells, bone cells, and fibroblasts. Regarding the biochemical mechanism, the action of PRP is achieved through platelets’ degranulation of α-granules that contain pre-synthesized growth factors. The secretion process starts about ten minutes after the beginning of the clot formation, and almost the entire amount (95%) is secreted within one hour. In the seven days following the initial clot formation, platelets synthesize additional growth factors and are successively degraded by macrophages that, from this phase onwards, orchestrate the healing process [36]. Haynesworth et al. [37] studied mesenchymal cells’ proliferation in correlation to the platelet concentration in vitro. They identified a dose–response effect beginning when the platelet concentration is 4–5 times greater than the baseline value. Similar results were obtained when studying fibroblastic proliferation and type I collagen production [38]. The minimum starting platelet count for healthy subjects, in order to achieve “therapeutic” PRP, is about 106 U/μL for a 6–7 mL aliquot.

An examination of premature platelet activation during the collection, processing, and storage of platelet concentrates may provide an explanation for PRP’s clinical inefficacy. The “platelet storage lesion” has been well documented in the literature, although the precise relationship between in vitro assays and in vivo platelet recovery and survival is yet to be established [39,40,41,42]. It has also been demonstrated that growth factor concentrations in PRP differ significantly based on the storage temperature, duration of storage, and method of activation. Kim et al. [43] posited that appropriate storage conditions and activation are important to optimize effects and ensure desired clinical outcomes.

Shen et al. [27] demonstrated platelet activation in the first day after preparation, which increased at 3 and 5 days. Despite the evidence of activation, PRP is often preserved for five days after preparation, with good clinical effects in dermatology and plastic surgery. However, there is limited data available on platelet activation under routine storage conditions and regarding utilization in oral and maxillofacial surgery.

In our department, to minimize the problem of the premature platelet activation and degranulation, PRP is routinely used within 24 h from its preparation.

However, to verify the platelet status in the PRP after 1, 6, and 24 h of storage, mimicking identical conditions of the PRP destined for the surgery room, six samples of 1 mL aliquots were prepared at one time and without interference from the main container. The sample preparation was performed after one hour of continuous gentle agitation of the initial whole amount of PRP on the same rotator so that the tubes contained a homogeneous quantity of PRP.

The storage of the samples, using Terumo^®^ connectors, guaranteed sterile conditions without any exposure of the platelet concentrate to the ambient atmosphere and enabled us to maintain the same atmospheric conditions as the main container. In this regard, our procedure is safer than that performed by Shen et al. [27], who collected PRP samples (3 mL) from the main container three times with a 16-gauge needle attached to a polypropylene syringe. This technique may affect the platelets’ status and influence the results regarding activation or degranulation.

On the contrary, the procedure adopted in this study enabled us to analyze the platelet status and functionality at standardized times and to observe the biological behavior of the platelets in the main bag.

Flow cytometry allows for the analysis of multiple cell surface antigens, and established protocols for the assessment of the platelet function in whole blood and in platelet-rich plasma gel have been introduced [44,45,46]. From the flow cytometric investigation, we demonstrated that the platelet concentrate was rich in platelets that were intercepted by CD 42 at 1, 6, and 24 h after preparation. The important decrease in the CD 42 positivity identified after gelification must be interpreted as a consequence of the entrapment of platelets in the fibrin network after gelification. After the first hour, more than 25% of platelets were positive for CD 62p, increasing to almost 40% of the whole platelet population. This finding demonstrated that during storage, platelets already started to expose CD 62p receptors, indicating the beginning of transmembrane signaling. This was confirmed by the amount of CD 63 membrane markers, which indicated that degranulation activity had started. At 24 h, this increase reached 80%. The analysis of these results must be performed carefully to avoid misinterpretations. Flow cytometry can link positivity to a specific marker independently based on the amount of the receptor exposed on the membrane. Using the ELISA test, it was possible to produce a quantitative definition of the growth factors that were already released during the storage of the platelet concentrate, which were potentially not available at the time of the PRP’s surgical use. In this regard, it is clear that an increase in platelet activation and degranulation (flow-cytometry) corresponded to an effective increase in the release of TGF-β (ELISA), but only about 20% of the total available TGF-β was effectively released over 24 h of PRP storage. From this, we can speculate that an abundant quantity of growth factors remains in the platelets and are therefore available at the surgical site, where the activation and degranulation will lead the maximum values after thrombin’s actions.

An increase in the release of TGF-β was then noted up to the 24 h mark, with a subsequent concentration reduction after gelification, bringing the levels back to values that were similar to those of the first hour. This decrease, as well as the significant decrease in the CD42 percentage, demonstrated that an amount of the TGF-β that has already released is re-englobed in the PRP during gelification and, if not already degraded, could support the healing of the surgical site.

Therefore, the trend observed in the storage phase suggests that use beyond 24 h would lead to progressive platelet activation and degranulation with a loss of valuable growth factors. Even if not statistically significant, there was a significant difference in the release kinetics of TGF-β between 1 h and 24 h and between 6 h and 24 h, thus reinforcing the idea that the clinical use of PRP within 6 h would be optimal.

In conclusion, our PRP preparation protocol ensures a good hemoderivative, and when the platelet concentrate produced is used with correct timing, it ensures adequate healing support.

## Figures and Tables

**Table 1:** Expression of platelet markers across experimental time points.

	1 h	6 h	24 h	Post-Gel	Sig *
CD42	79.1 ± 20.05	90.2 ± 23.63	97.6 ± 25.31	11.9 ± 2.1	*p* < 0.005
CD62p	25.71 ± 25.60	32.25 ± 30.85	39.21 ± 27.48	Not dosable	NS
CD63	59.86 ± 32.75	62.99 ± 31.02	80.43 ± 16.94	Not dosable	NS

* Friedman test; NS = not significant.

**Table 2:** Release percentage and mean concentration of TGF-β in supernatants at different time points.

	1 h	6 h	24 h	Post-Gel	Sig *
% release = SN/TOT	8.4 ± 7.3	14.8 ± 8.7	19.9 ± 5.0	8.6 ± 3.7	NS
TGFβ ng/mL (mean ± SD)	18.36 ± 12.41	21.96 ± 13.15	29.45 ± 14.24	15.52 ± 14.24	NS

* Friedman test; NS = not significant.

## Data Availability

The original contributions presented in this study are included in the article. Further inquiries can be directed to the corresponding author.
